# Predetermined sex revealed by a female transient gut in non-feeding larvae of *Osedax* (Siboglinidae, Annelida)

**DOI:** 10.1186/s13227-025-00251-9

**Published:** 2025-08-26

**Authors:** Alice Rouan, Norio Miyamoto, Katrine Worsaae

**Affiliations:** 1 Marine Biological Section, Department of Biology, University of Copenhagen, Universitetsparken 4, 2100 Copenhagen Ø, Denmark; 2 X-STAR, Japan Agency for Marine-Earth Science and Technology, Yokosuka, Japan

**Keywords:** Sex determination, Sexual dimorphism, Rudimentary gut, Lecithotrophic larvae, Trochophore, Vascular system

## Abstract

**Background:**

Within the symbiont-hosting Siboglinidae (Annelida), *Osedax* stands out as the sole genus capable of degrading bones and displaying pronounced sexual dimorphism (except *O. priapus*). While macroscopic, gutless females feed on whale falls with their symbiont-housing “roots”, males are microscopic and non-feeding. Yet, embryos and larvae look identical, and sex is suggested to be environmentally determined, i.e., larvae metamorphose into females on bare bone or into males when finding an adult female.

**Results:**

However, we here describe a transient gut present in half of the late larvae and in juvenile females of *O. japonicus*. We confirm the gut-carrying larvae as being females from sex-specific in situ gene expression. Moreover, morphological evidence coupled with differential gene expression indicate that the ‘non-feeding’ transient gut may pattern the vascular system and/or act as a gas-exchange surface in juvenile females, before their branchial appendages develop.

**Conclusions:**

The transient gut of *O. japonicus* females reveals a genetic sex determination. Proposedly homologous across siboglinids, this vestigial gut is suggested to function in organ patterning and/or for gas-exchange during development of the gutless adult.

**Supplementary Information:**

The online version contains supplementary material available at 10.1186/s13227-025-00251-9.

## Background

Adult members of Siboglinidae (Annelida) are tube-dwelling, gutless worms nutritionally dependent on endosymbiotic bacteria [1–3]. The ability of these organisms to establish symbiotic relationships with diverse bacterial species (including heterotrophs, methanotrophs, and thiotrophs) has allowed them to inhabit extreme marine environments, such as hydrothermal vents, cold seeps, and whale falls [4–8]. Among Siboglinidae, only adult representatives of the *Osedax* genus demonstrate the ability to feed on bones and exhibit extreme sexual dimorphism [2, 9, 10]. *Osedax* females generally possess four palps used for respiration, a trunk surrounded by a mucus holster, and a posterior end, named root system, penetrating the bone [11–17]. They host dynamic and heterogeneous assemblages of bacteria, including heterotrophic Oceanospirillales gammaproteobacteria, within bacteriocytes in a continuous tissue that extends through their root system [5, 18–20]. The males are dwarfs, largely resembling the non-feeding metatrochophore larvae and kept in harems, accumulating over time within the mucus holster of the female [10, 21–23]. Such extreme sexual dimorphism is only found in a few other annelids [24, 25], and among these, the dwarf males of *Bonelia viridis* are sexually determined by female hormonal cues [26]. These observations, together with the relative size and morphological similarities in embryos and larvae, led to the hypothesis of an environmental sex determination mechanism in *Osedax* [10, 27]. It was postulated that larvae colonizing a bare bone would become females, whereas only the presence of already settled females would induce male development, potentially through hormonal control, thereby increasing survival in an unstable habitat.

*Osedax* females release fertilized eggs from the oviduct, which runs externally on the dorsal side of the trunk and exit between the four palps [12, 13, 21]. The egg develops into a free-swimming, metatrochophore larva, with an apical organ, a three-ringed ciliary prototroch, a posterior telotroch and a terminal tuft [21, 22, 28]. The competent metatrochophore was in *O. japonicus* found to possess two parallel ventral trochs (paratroch1 and 2) and 16 chaetae after 5 days of development [15, 22, 28]. Like other siboglinids, *Osedax* larvae are lecitotrophic with a rapid development and limited larval growth [21, 22, 29–35].

Adult siboglinids lack a gut; however, a mouth opening, and a transient ciliated digestive tract have been documented in larval and juvenile stages. In Vestimentifera, unassigned larvae (collected in proximity to a *Riftia pachyptila* colony) and larvae of *Ridgeia* sp. were observed to possess a ciliated through-gut with a ventral mouth opening, located posterior to the prototroch and appearing in the early metatrochophore stage [31–33, 36]. In Frenulates, a mouth opening originating from the blastopore was first described for *Oligobrachia mashikoi* [37] and ciliated cells forming a primitive gut were subsequently reported for *Siboglinum* sp*.* larvae [7, 38–40]. The comprehensive description of *S. poseidoni* gut development [30] revealed its initiation at the metatrochophore stage (chaetae, prototroch, telotroch, and ventral ciliary field) and its growth until a late juvenile stage, although no anus was described. In addition, the presence of a mouth and a gut with a lumen, but no anus, was reported in trochophore larvae of *O. mashikoi* exhibiting a prototroch and a telotroch [37, 38]. For a time, symbionts were thought to be acquired through the midgut region of the transient ciliated tract [41]. However, this was disproved by Nussbaumer et al. [42], showing an epidermal acquisition, when following the migration of bacteria in different stages of vestimentiferans larvae. In adult *Osedax*, symbionts are acquired horizontally through the ‘root’ epidermis, burrowed in the vertebrate bone [5, 18, 19]. Although exact acquisition timing and mechanisms was limited by the larval stage availability, previous FISH study has shown that *O. japonicus* symbionts are not present until the juvenile stage (4-day post-induction (dpi) females, [15]). No morphological differences were reported during larval development in the few *Osedax* species studied at that stage [15, 21, 43], except for a brief mention of a potential gut structure in the more detailed developmental study by Worsaae et al. [22].

In other annelids, the gut has been studied at the molecular level using conserved gut markers, such as Forkhead A (*FoxA*), Goosecoid (*Gsc*), GATA transcription factors (*Gata*), Brachyury (*Bra*) and Wingless (*Wnt*) [44–55]. *FoxA* is usually expressed ventrally in the stomodeum and the foregut, and dorsally in the hindgut and the anal opening [46, 47]. Some species present an early expression in the gastrula stage such as *Hydroides elegans* [44], an extension of the expression in the midgut (*Owenia fusiformis*, [52]), or additional ectopic expression (developing parapodial area, ventral midline epithelium, lateral cells in the episphere ventral neuroectoderm) as in the nectochaete of *Alitta virens* and *Platynereis dumerilii* [51]. *Gsc* is another highly conserved gut marker, showing larval expression in the oral ectoderm and the stomodeum (*O. fusiformis*, *P. dumerillii*) [45, 55] as well as in the foregut and in anterior neural cells (*Capitella teleta*) [48]. In annelids the paralogs of *Gata4/5/6* are involved in the mesoderm or endoderm layer formation and are expressed in the midgut of several species, such as *O. fusiformis*, *T. lageniformis*, *Chaetopterus* sp*. *and* C. teleta* [46, 47, 52]. However, *P. dumerilii* only has one ortholog of *Gata456* expressed in the endomesoderm [49]. Finally, several *Wnt* (*Wnt1*, *Wnt4* or *WntA*) have been reported to have a ventral expression in the larval gut region [53, 54].

To elucidate the presence and nature of a variable morphological character, such as a ciliated tract (‘gut’) in *Osedax japonicus* larvae, we used a combination of immunostaining and whole mount in situ–hybridization chain reaction (WMIS–HCR) [56]. Our work is the first to show morphological differences among individual larvae of the same stage in an *Osedax* species and to identify correlated genetic markers to document the larval sex and presence of a transient ciliary gut. Using available genomic resources and adding new data to an individual stage transcriptome [28, 57], we shed light on the genetic basis of the transient gut and provide evidence of early sexual differentiation in *O. japonicus* larvae.

## Methods

### Animal collection and fixation

We used a laboratory culture of a non-endangered marine annelid species, *Osedax japonicus* (see [15, 43]). The females were kept at 10 °C on vertebrate bones (see [15, 43]). Fertilized oocytes were dissected out from the mucus sampled around the female and kept at 10 °C in filtered artificial sea water (ASW). Collection time was registered, and samples were fixed consecutively. Metamorphosis was induced by exposing 5-day-old competent larva to a sea bream scale (*Pagrus major*) previously washed and incubated in bacterial *Oceanospirillales sp.* culture.

Animals were fixed using a previously described protocol [22]. Animals were anesthetized in 7% magnesium chloride (MgCl2) before being fixed in 4% paraformaldehyde (ThermoScientific, 043368.9 M) in phosphate-buffered saline solution (PBS) with 7% sucrose for 1-2 h at room temperature (RT). Samples were rinsed in 7% sucrose PBS 6 times before being stored with 0.05% NaN3 at 4 °C for F-actin staining or gradually dehydrated to 100% MeOH and stored at − 20 °C for HCR–IF.

### RNA extraction

Samples were extracted as described in [28]. We used the previously generated data set (BioProject PRJNA1088276) composed of RNA-bulk sequence of 5 individuals of early embryo, 1-day-old larva (1D), 2D, 3D, 4D, and 10 individuals of competent larva (5D and 6D) (see 22 for staging), as well as 3 individual replicates for the early adult stage (1dpi for female and 6–8 h post-induction (hpi) for males) and the late adult stage (3-week post-induction (wpi) females, 4dpi for the males) [28]. To that were added, based on the same staging, 5 individuals of early adult male and early adult female, 4 late adult male individuals and 2 late females (Table S4). Briefly, those samples were fixed in RNAlater and stored at − 80 °C. RNA extraction was performed using the ultra-low input SMART-Seq® HT Kit (Takara Bio USA, Inc.). Prior to being transferred to lysis buffer, each individual was rinsed with cold and sterile RNAlater. Samples were stored at − 80 °C for up to 5 days or processed following the kit protocol for extraction and cDNA synthesis. Quantity of cDNA was measured using a Qubit® dsDNA HS (High Sensitivity) Assay Kit, and quality using an Agilent 2100 Bioanalyzer (Agilent Biosciences) with the high sensitivity DNA kit. We used 1 ng of cDNA to prepare dual-indexed libraries with the Nextera® XT DNA library preparation kit (Illumina). Genewiz™ Agenta Life Sciences (Leipzig, Germany) performed 150 bp paired-end Illumina sequencing on the multiplexed libraries on a NovaSeq 6000 platform. Samples sequences are deposited at NCBI Sequence Read Archive (SRA) under the BioProject (PRJNA1217611).

### Immunostaining

To stain both the ciliary and muscular system to highlight e.g., gut and heart body, we performed immunostaining as described in [22]. Specimens stored in PBS–NaN3 were rinsed two times in PBS with 7% sucrose before a 4–6 h pre-incubation in 5% PTA (1X PBS, 5% Triton-X, 0.25% BSA, 7% sucrose) followed by a 48 h incubation on a rocking table at RT in primary antibodies mixed 1:1 with a final concentration of 1:800 [monoclonal mouse anti-acetylated α-tubulin (SigmaAldrich, T6793)] in 0.1% PTA (1X PBS, 0.1% Triton-X, 0.25% BSA, 7% sucrose). Rinses in 0.1% PTA of 15–30 min 4 times were applied before 24 h incubation on a rocking table in secondary antibody [1:800 final concentration goat anti-mouse Cy5 (Jackson ImmunoResearch, 115–175-062)] and 33 uM of Alexa Fluor 488-labelled phalloidin (INVITROGEN, A12379). Samples were then washed four times for 30 min each in PBS with 7% sucrose and gradually raised to 100% Vectashield (VECTOR LABORATORIES, Burlingame, USA) mounting media with DAPI before being individually mounted on slides.

### HCR coupled with IF

Coding sequences were retrieved from the transcriptome data set and used to generate probe pairs with the In Situ* Probe generator* software [58] and ordered from DNA Technologies (IDT) (Table S2). HCR were performed using the HCR RNA–FISH buffers and amplifiers (B1–488, B2–546, and B3–633) from Molecular Instruments (LA, USA). Samples stored in MeOH were rehydrated in decreasing concentrations of MeOH (95%, 75%, 50%, and 25%) and rinsed twice in PBT (1X PBS, 0.1% Tween). The permeabilization step was carried out for 1 min at RT in 0.1 mg/mL Proteinase K in PBT, followed by a quick PBT wash and a 15 min incubation in 2 mg/mL glycine in PBT on ice. Prior to a 30 min post-fix in 4% PFA on ice sampled were rinsed twice in PBT on orbital shaking. Samples were rinsed in PBT on ice 5 × 5 min, pre-incubated in 1:1 PBT/hybridization buffer for 10 min at RT and pre-hybridize for 1 h at 37 °C in hybridization buffer. The samples were incubated with 4 pmol of each probes set overnight at 37 °C. Therefore, four steps of 15 min washes using the hybridization wash buffer were performed, followed by 2 × 5 min in 5 × SSCT (5xSSC, 0.1% Tween), 2 × 5 min in PBT and a 30 min pre-incubation in amplification buffer. Each hairpin (h1, h2) for each (B1, B2, B3) amplifier, using 15 pmol each, was previously denatured for 90 s at 95 °C and then cooled over 30 min to room temperature prior mixing. Primary antibodies were added at this stage in concentration of 1:800 for α-tubulin (monoclonal mouse anti-acetylated α-tubulin, SIGMA, T 6793). Hairpins and primary antibodies were incubated for 48 h on a rocking table at 26 °C. Afterwards samples were washed 2 × 5 min in 5 × SSCT and 3 × 10 min in PBT and incubated over 24 h/48 h with secondary antibody in PTA 0.1% (1:800, goat anti-mouse Cy5, Jackson Immuno-Research 115-175-062). Samples were washed 3 times in PBS 7% sucrose and brought gradually to 100% DAPI-vectashield (VECTOR LABORATORIES, Burlingame, USA) before being mounted individually. Slides were kept at − 20 °C and scanned in the 2 weeks following mounting. Probes for GataA1, Wnt4 and FoxA4 did not show significant results (data not shown).

### Confocal imaging

The slides were scanned on an OLYMPUS IX 81 inverted confocal microscope with a Fluoview FV-1000 system using a 60X oil immersion objective. Acquired z-stacks were analyzed with Imaris viewer 10.0 (Bitplane Scientific Software) and maximum intensity z-projections were created from cropped or full z-stacks. The ratio of the presence or absence of a gut in larval and adult stages was assessed by looking at anti-acetylated-α-tubulin stained scans (Table S1). Measurements of the mouth opening, gut and length of the animal were performed and averaged on 3 replicates and done with ImageJ (Fiji v1.54f) (Table S1). Drawings and figures were made and assembled on Inkscape (v1.3.1). To ensure visibility of the signal some stacks from scans were virtually cropped and specified in legend; full scans used for the figures are available on ERDA.

### Transcriptome analysis

Quality of the newly sequenced sample was assessed using FastQC (v.0.11.9), reads were trimmed with Trim_galore (v0.6.4) using paired-end library and adapter sequences detection parameters, the de novo reference was assembled with Trinity (v2.15.1) and filtered for prokaryote contaminants using ncbi-magicblast (v1.7.1) against the prokaryote genome reference databases. ORFs were identified using TransDecoder (v5.5.0) and signal peptide annotation was performed on the translated peptide file from cds using signalp (v6.0). The annotation was done using Trinotate (v.4.0.2). The reads were mapped on the new de novo assembly and quantified with Kallisto (v0.46.0). Computation was performed on the Danish National Life Science Supercomputing Center-Computerome 2.0 (www.computerome.dk). Downstream data analysis was conducted on R (v4.3.1) using Rstudio, annotations were imported using *TrinotateR* and Kallisto reads were loaded using *tximport* [59]. The differential gene expression analyses were conducted using the *edgeR* [60] and *limma* [61] packages. Data were averaged based on developmental stage and sex, using the *limma* package. GO-Terms enrichment analyses were conducted using *TopGO* package. The subset of overexpressed genes (762 genes) in females (4D/5D/juvenile) against same male stages was used to perform a weighted correlation network analysis (WGCNA) on all the stages using the *WGCNA* [62] package, the soft-thresholding power was estimated to eight. Data were visualized and plotted using *ggplot2*.

### Orthology analysis

Sequences were retrieved from different sources, Gsc, FoxA and WntA, 1 and 4 were retrieved from annotated genes from NCBI and from martinduranlab.com genome annotation repository, GATA genes data set was retrieved from [47]. Sequences and accession numbers (when available) are compiled in Table S3. Sequences were aligned using *MUSCLE* and maximum likelihood analyses were conducted on *Geneious*. Trees were generated and rooted using IQTree [63], using 1500 ultrafast bootstrap replicates, the best-fit model for each alignment was as followed: [JTT + I + G4] for *Gsc*, [*Q.insect* + *F* + *I* + *G4] for FoxA,* [VT + F + I + G4] for *Gata*, and [LG + I + G4] for *Wnt* (Fig. S3).

## Results

### Timing of gut development

**Fig. 1:**
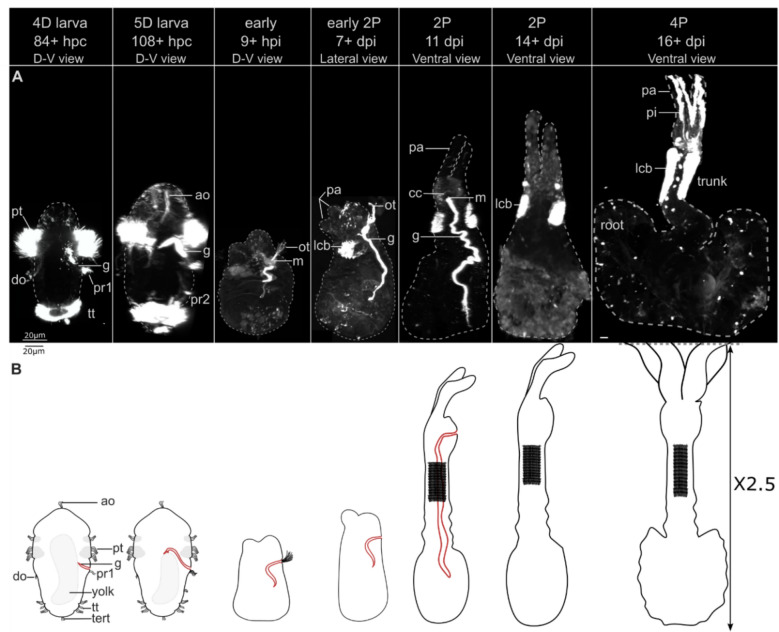
*Osedax japonicus* gut development from the 4-day-old (4D) larva to the 2 palps (2P) juvenile female stage. **A** Confocal laser scanning microscopy (CLSM) of anti-acetylated α-tubulin (*white*) stained cilia showing lateral views of 4D and 5D larvae, early metamorphosed females, and early 2P females, ventral views of later 2P stages and mature adult 4P stage. Lateral projections were virtually sectioned to show the gut in larval stages, **B** scaled corresponding drawings in lateral view (dorsal to the left, ventral to the right); the 4P drawing is reduced 2.5× in size. *ao* apical organ, *cc* circumesophageal connectives, *D–V* dorso-ventral, *do* dorsal organ, *dpi* days post-induction, *g* gut, *hpc* hours post-cleavage, *hpi* hours post-induction, *lcb* lateral ciliary band, *m* mouth, *ot* oral tuft, *P/pa* palp, *pi* pinnules, *pr1-2* paratroch 1–2, *pt* prototroch, *tert* terminal tuft*, tt* telotroch. Scale bars: 20 µm

We conducted confocal laser scanning microscopy (CLSM) of 143 acetylated-α-tubulin labelled *O. japonicus* specimens of different stages (Table S1). Larvae from 0-day-old (0D) to 3D had no evidence of a gut (*n* = 22). We found a highly ciliated internal tract in almost 50% of the 4D (11 out of 24) and 5D (21 out of 44) larvae, generally characterized by the presence of two ventral ciliary bands, the paratroch1 and the paratroch2 (Fig. 1A, B). We also observed that gut-like structure in 100% of the juvenile female (2-palps (2P) stage), including the 11dpi (*n* = 15) (Fig. 1A, B). Both adult females (*n* = 3), and early (*n* = 13) and late (*n* = 3) male stages investigated lacked such gut structure (Table S1).

In the 4–5D female larvae, the short ciliated ‘gut’ opens ventrally, forming the mouth, posterior to the prototroch and just anterior to the paratroch1 (Fig. 1A, B). The gut diameter is only 4.5 µm (sd = 0.4, *n* = 3) and a lumen could not be distinguished with CLSM (Fig. 1A, Table S1). In the 5D stage, it extends internally and anteriorly from the mouth before bending 180 degrees to terminate centrally in the post-trochal part (no posterior anus). On average the gut accounts for 29% of the total larval length (5D larvae, *n* = 3) (Fig. 1A, B, Table S1). The gut orientation changes to extend directly centro-posteriorly from the mouth during and post-metamorphosis. A bundle of cilia emerges from the mouth (oral tuft) in the early metamorphosed stage, which persists until the development of the first two palps and the lateral ciliary band (Fig. 1A, B). The gut keeps extending centro-posteriorly, elongating as the trunk region grows and reaching up to 69% of the 2P-female’s total length (*n* = 3) (Fig. 1A, Table S1). At the 2P stage, the dorsal palps are fully developed but not yet covered with ciliated pinnules as observed in the 4-palps (4P) females (Fig. 1A). The ciliated gut is resorbed between 11 and 14dpi, before the development of the two ventral palps, as it is not visible in the 14 + dpi stages (Fig. 1A). Finding a nearly 1:1 proportion of gut/gutless 4D and 5D larvae, while early males are always gutless and early females always have a gut, suggests that 4D/5D larvae with gut are female larvae, and that the other half, without gut, are male.

Adult *O. japonicus* males do possess other ciliary ducts that only appear post-metamorphosis. However, the position and opening of these differ from the female ‘gut’ structure. Similar to what has been found in other species of *Osedax* these comprise two lateral ciliary tracts (potential seminal ducts) that fuse into a centro-dorsally bending *vas deferens* that opens dorsally, posterior to the prototroch [23, 64].

### Early larval differential gene expression between sexes

**Fig. 2:**
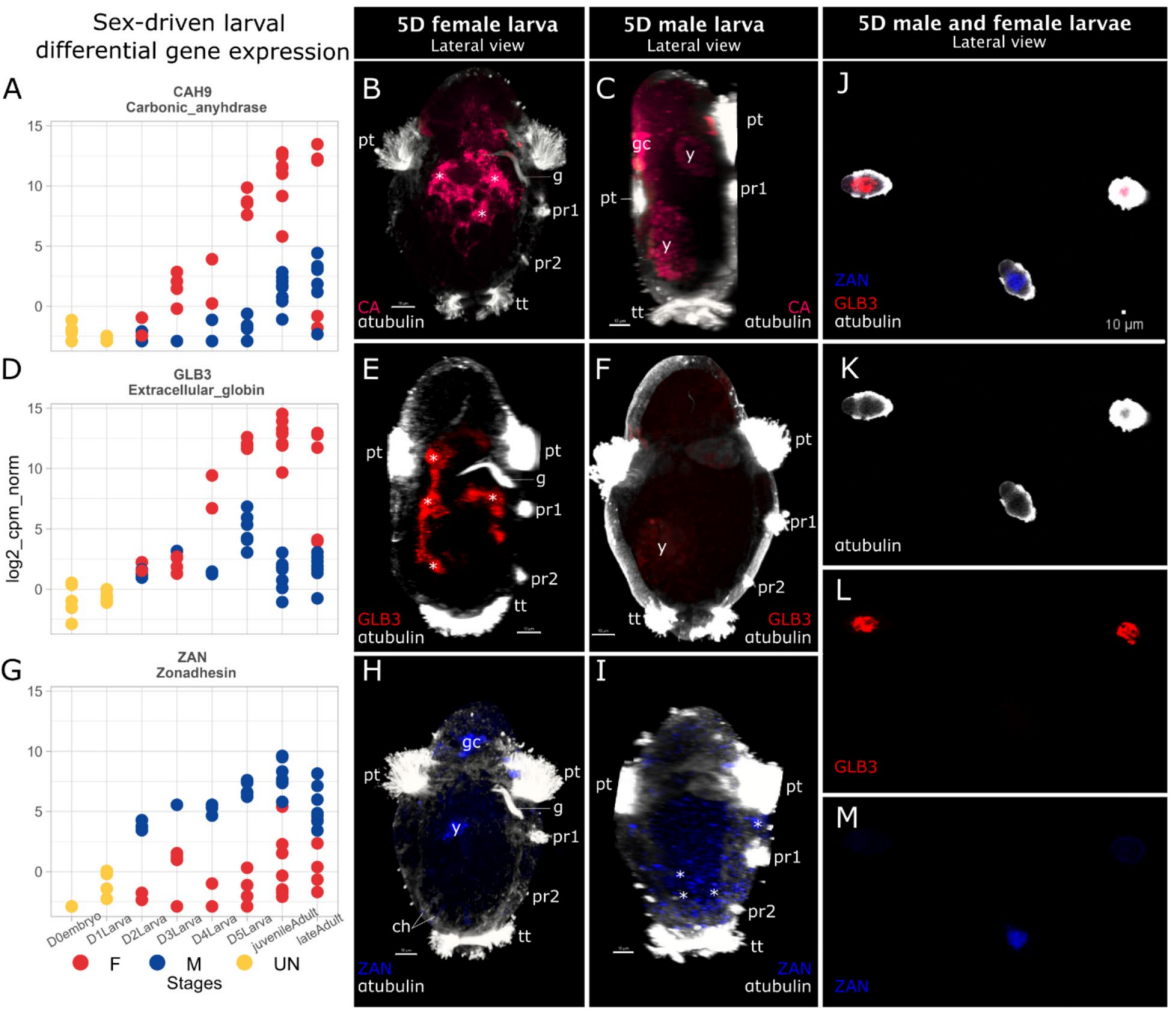
Differential gene expression between sexes in larval stages of *O. japonicus*. **A**–**D**–**G** Log2 scaled normalized gene expression (cpm) of carbonic anhydrase (*CAH9*), extracellular globin (*GLB3*) and zonadhesin (*ZAN*). Individual dots show individual samples of undetermined sex (*yellow*), female (*red*), male (*blue*), **B**, **C**–**E**, **F**–**H**, **I** dorso-ventral (left to right) view of maximum intensity z-projection of whole mount in situ-HCR CLSM scans showing *CAH9* (*dark pink*), *GLB3* (*red*) and *ZAN* (*blue*) coupled with acetylated α-tubulin-like immunoreactivity (*white*) in female (*left*) and male (*right*) 5-days-old (5D) larvae. Lateral projections were virtually sectioned to remove prototroch signals for better display of intended structures. **J** WMIS–HCR CLSM scans of 5D larvae siblings showing **K** anti-acetylated α-tubulin-like immunoreactivity (*white*) **L** *GLB3* (*red*) expression and **M** *ZAN* (*blue*) expression. *5D* 5-day-old, *ch* chaetae, *g* gut, *gc* glandular cell, *pr1-2* paratroch 1–2, *y* yolk. Asterisks (*) highlight signals. Scale bars: 10 µm

Looking for genetic evidence of an early sexual differentiation we took advantage of an individual transcriptome data set from Tilic et al. [28] and complemented it by generating 16 new transcriptomes of early and late adults (Table S4). We identified genes highly and specifically expressed in female or male adults (Fig. S1). From those differentially expressed genes (DEG) between sexes in adults we identified genes already expressed in different pools of larvae (Fig. 2A, D, G). About half of the larval transcriptomes had high expression levels of three genes specifically expressed in adult females: extracellular globin B3 (*GLB3,* Ojap_20494_c0_g1), a carbonic anhydrase (*CA,* Ojap_18712_c10_g1), and an oestrogen receptor (*ER,* Ojap_10505_c0_g1) (Figs. 2A–D, S1). The other half of the larval transcriptomes had high expression levels of a male specific gene, zonadhesin (*ZAN,* Ojap_2409_c0_g1) (involved in the acrosomal fusion), suggesting that these were male larvae and the other pool female larvae (Fig. 2G). We confirmed the genetic female identity of 5D larvae exhibiting a gut using probes against *GLB3*, *CA* and *ZAN* and performing an HCR–WMISH, coupled with acetylated-αtubulin immunostaining (Fig. 2). The high background signal of the yolk and of pigmented, potentially glandular, cells found in-between the rows of prototroch cells is highlighted using B2 and B3 amplifiers alone as negative control (Fig. S2). Our results revealed both *GLB3* and *CA* signals in only the 5D larvae having a ciliated gut (Fig. 2B, C, E, F, Table S1, respectively, *n* = 11/11 and *n* = 8/8 of 5D with gut). To confirm the genetic male identity, we used probes against *ZAN*. Apart from the yolk and prototroch glandular cell background, no signal was detected in 5D larvae with a gut (*n* = 14/14) and signal was found in 5D lacking a gut (*n* = 3/4) (Fig. 2H–M, Table S1). We further confirmed the sex ratio (on average 1:1) through repeated staining experiments using larval batches from individual mothers (Fig. 2J–M, Table S1). Having experimentally confirmed the sex specificity of the genes, we could now assign a sex to each larval transcriptome based on the expression level of these four genes, starting from the 2-day stage (Fig. 2).

### Gut/ventral markers HCR expression pattern

**Fig. 3:**
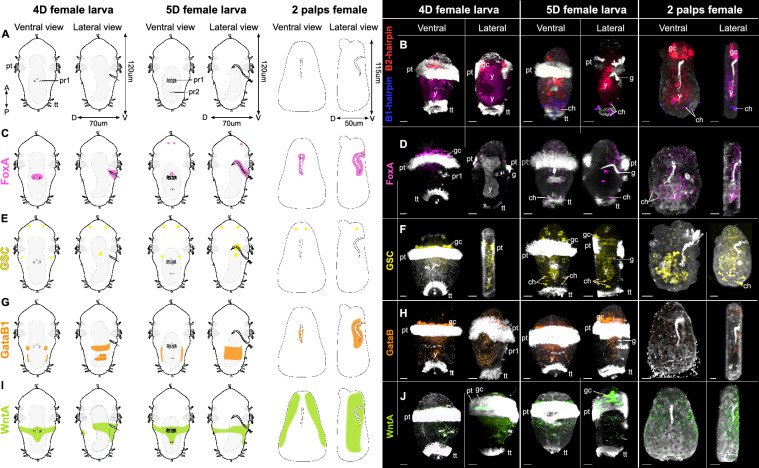
Ventral and lateral views of temporal expression pattern visualized by whole mount in situ-HCR coupled with anti-acetylated α-tubulin immunostaining (*white*) in 4-day-old (4D), 5D female larvae and early 2 palps (2P) females. **A** Scaled drawings of female ventral and leral view, **B** HCR negative control using only B1-(*blue*) and B2-(*red*) hairpins highlighting the yolk, pigmented prototroch cells and chaetae background, **C**, **D** drawing and HCR of *FoxA* (*pink*) expression pattern, **E**, **F** drawing and HCR of Goosecoid (*GSC*) (*yellow*) expression pattern, **G**, **H** drawing and HCR of *GataB1* (*orange*) expression pattern, **I**, **J** drawing and HCR of *WntA* (*green*) expression pattern. **C**–**E**–**G**–**I** Drawings represent the conserved signal found across individuals of the same stages. Blue dotted lines highlight the probes’ signal. Lateral projections were virtually sectioned to remove prototroch signals for better display of intended structures in lateral views. *4D/5D* 4/5-day-old, *ch* chaetae, *D* dorsal, *gc* glandular cell, *pt* prototroch, *pr1/2* paratroch 1/2, *tt* telotroch, *V* ventral, *y* yolk. Scale bars: 15 µm

To further describe the development of the transient gut we examined potential gut/mouth marker genes. Orthology analysis allowed us to shed light on the presence of *WntA* (Ojap_5228_c0_g2), misannotated *Wnt4*, not only in *O. japonicus* transcriptome but also in the genome of *O. frankpressi* (Fig. S3, [57]). In addition, we uncovered *FoxA* (Ojap_12147_c0_g2), *Gsc* (Ojap_1633_c3_g1) and two Gata assigned to GataA (*Gata1/2/3*) and GataB (*Gata4/5/6*) and accordingly named *GataA1* and *GataB1* (Ojap_10785_c0_g1) (Fig. S3). We used the amplifiers B1 and B2 alone as negative control to highlight the high background of the yolk and of pigmented, potentially glandular, cells found in-between the prototroch cells, present in larvae and 2P females, as well as the autofluorescence of the chaetae (Fig. 3A, B).

Larval gene expression analyses showed *FoxA* to mainly be expressed in female (Figs. S3, S4) and HCR–WMISH experiments localized *FoxA* ventrally in the oral ectoderm and along the gut from the 4-days-old (4D) to the early adult 2-palps (2P) female stages (Figs. 3C, D, S5). Two additional apico-ventral cells were labelled at the 5D stage (Fig. 3C, D). *Gsc* is expressed at the highest in the 1D stage, and in both sexes in the 2D to 4D stages (Fig. S3). *Gsc* is mainly present in two cells on the dorsal side of the apical plate, followed by two symmetrical ventral cells in the anterior end of 4D and 5D female larvae (Fig. 3E, F). In addition, a few cells are labelled internally on the latero-ventral side, between the prototroch and the paratroch1 at the 4D stage (Fig. 3E, F). At the 5D stage those few cells are found more medioventrally, closer to the gut, as well as additional cells surrounding the gut at its curvature (Figs. 3E, F, S5). Gene expression of *GataB1* starts from the 1D stage in both sexes and remains at a high expression level in female larvae (Fig. S3). At the 4D stage *GataB1* expression pattern is located on each lateral side in two to three distinct transverse, mesodermal bands that fuses into a broader band in the female 5D larvae (Figs. 3G, H, S4). Interestingly, *GataB1* is expressed in the ciliated gut cells in the 2P female but not in the oral ectoderm (Fig. 3G, H). *WntA* was not specifically expressed in females (Fig. S3) and the in situ staining revealed both a ventral ectoderm, around the mouth, and dorsal expression pattern that join laterally in 5D female larvae while being restricted to the ventro-lateral ectoderm in juvenile 2P females (Figs. 3I, J, S4).

### Horizontal transfer of symbionts in juvenile females

**Fig. 4:**
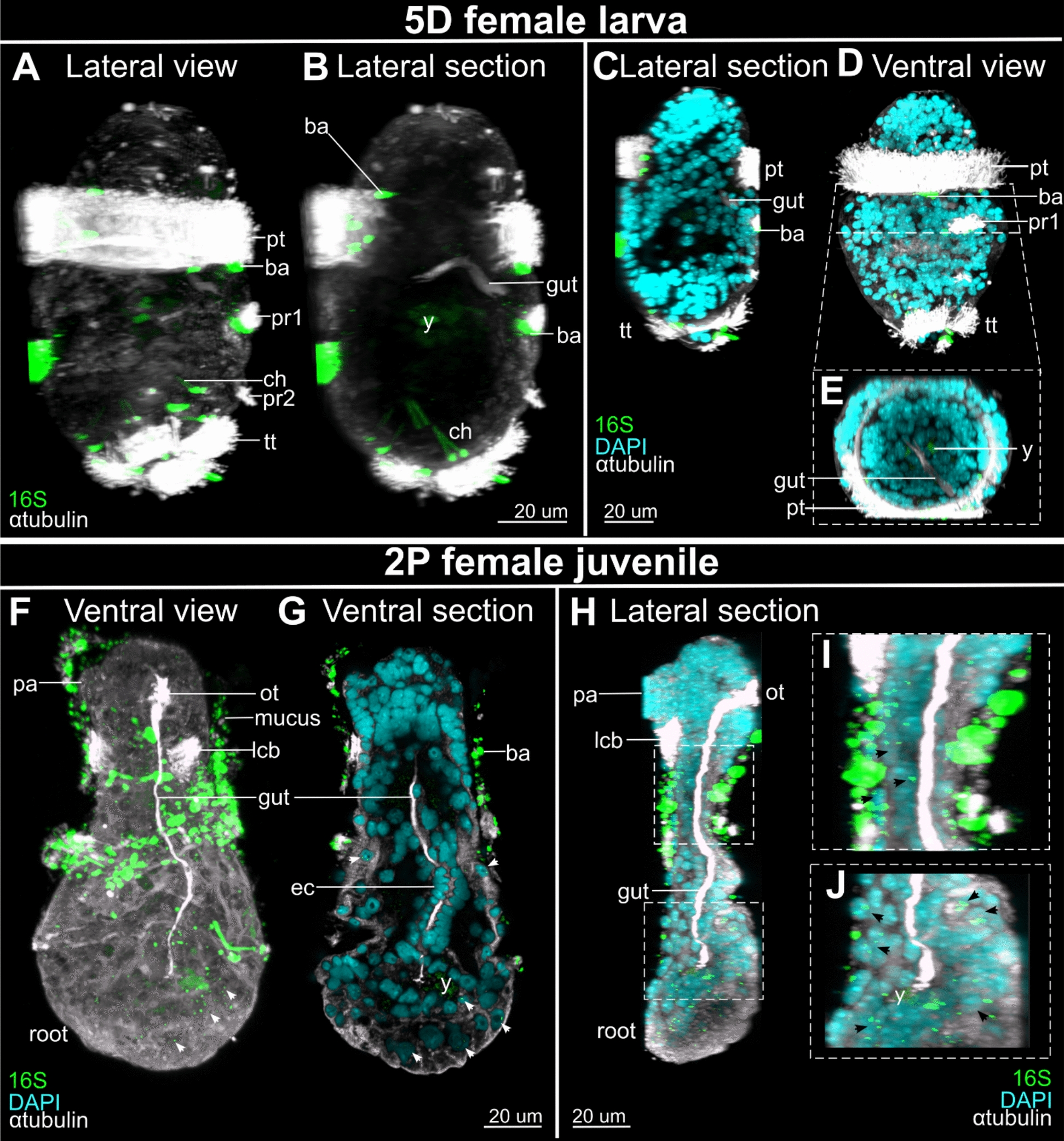
Ventral views and lateral views (ventral to the right) of symbiont localization by whole mount in situ-HCR in *O. japonicus* 5-days-old (5D) female larvae (**A-E**) and 2-palps (2P) females (**F-J**). **A** Lateral, maximum z-projection only showing bacterial aggregates externally among cilia, using 16S probes (*green*). **B** Mid-sagittal view, achieved by virtually removing outermost lateral sections, showing lack of bacterial signal in or near the gut. **C** Mid-sagittal view, showing nuclei signal (DAPI, *cyan*). **D** Ventral, maximum z-projection only showing external bacterial aggregates. **E** Cross section of gut region showing no bacterial signal in the endoderm gut cells. **F** Ventral, maximum z-projection with bacterial 16S probe signal externally, trapped in mucus, and internally, within cells of the root and trunk (arrowheads).**G** Sub-ventral view, achieved by virtually removing outermost ventral sections, showing lack of bacterial signal in gut. **H** Mid-sagittal view of multiple sections, showing similar signal as F. **I-J**, close-up of H showing external bacterial aggregates in mucus and internal signal in lower trunk (I) and root (J) tissue. *ba* bacteria aggregates, *ch* chaetae, *ec* endoderm gut cell, *lcb* lateral ciliary band, *ot* oral tuft, *pa* palp, *pr1-2* paratroch 1–2, *pt* prototroch, *tt* telotroch, *y* yolk. Arrowhead highlight signals. Scale bars 20 µm

*Osedax* adult females have been shown to acquire their intracellular symbionts horizontally through the epidermis of the posteriormost trunk region [5, 15, 18, 19]. Yet, to test for a potential function of the transient gut in early symbiont acquisition we exposed 5D competent metatrochophore to a bacterial culture of *Oceanospirillales*. Bacteria were labelled using HCR designed *Oceanospirillales* 16S probes. No bacterial 16S signal was observed in the 5D larval gut dermis, gut cilia, nor around the mouth opening (Fig. 4A–E). Some bacterial bundles, highly labelled with the 16S probes, were stuck within external ciliary bands, such as the prototroch, showing the presence of bacterial aggregates on the epidermis (Fig. 4A, B). At the 2P stage most of the signal was found in the mucus surrounding the females, but also within the roots and the trunk (Fig. 4F–J), further supporting the presence of *Oceanospirillales* symbionts in juveniles and adults as well as their absence in the larval and juvenile transient gut (Fig. 4F–J).

### Dorsal blood vessel ‘heart’ line the transient gut

**Fig. 5:**
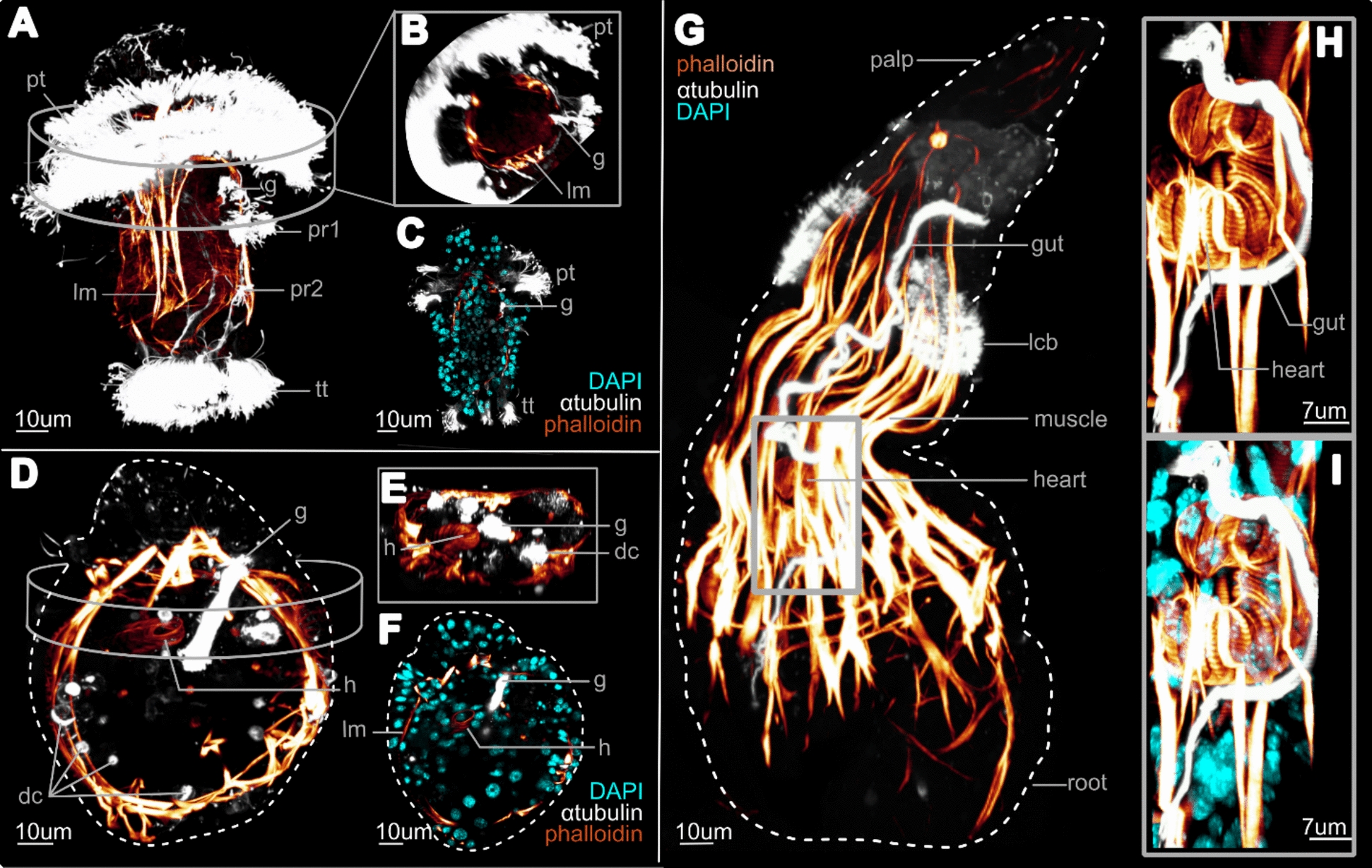
Ventro-lateral views of *O. japonicus* CLSM phalloidin stain (*red–yellow gradient*) showing the heart development relative to the gut (anti-acetylated α-tubulin stain, *white*) and nuclei (DAPI, *cyan*). **A** 5-days-old (D) larva showing the position of the gut relative to the musculature (*red–yellow gradient*). **B** Mid-anterior trans-section view. **C** Dorso-ventral (left–right) longitudinal section showing actin staining near the gut. **D** Early metamorphosed female showing the elongation of the gut medioventrally. **E** Medioventral trans-section showing the heart and gut proximity. **F** Dorso-ventral (left–right) longitudinal section showing the cellular contact between heart and gut. **G** 2-palps (2P) female stage showing the longitudinal elongation of the gut with the trunk and its direct contact with the heart body. **H**, **I** Enlargement of the heart body. **C**, **D**–**F**, **G**, **H**, **I** Lateral stacks were virtually removed from maximal z-projection to show the heart structure and removed longitudinal muscle. Abbreviations: *dc* degenerated cilia,* g* gut*, h* heart, *lcb* lateral ciliary band, *lm* longitudinal muscle, *pa* palp, *pr1-2* paratroch 1–2, *pt* prototroch, *tt* telotroch. Scale bars: **A**–**G** 10 µm and **H**, **I** 7 µm

The musculature and blood vascular system of female *Osedax* has been extensively examined and myogenesis was recently described for the *O. japonicus* larva [13, 14, 20, 22, 23]. However, none of these studies reported the position of the gut relative to the developing blood vascular system in female juveniles. Here we re-examined the female metatrochophore and juvenile musculature to focus on the position of the highly muscularized posterior part of the dorsal blood vessel, referred to as ‘heart’, relative to the transient gut (Fig. 5). The formation of the vascular system likely onsets early during the metamorphosis of the female larva, as it is absent in the 5D female larvae but already developing in early metamorphosing females (Fig. 5A–F). Both the gut and heart extend internally to meet at their respective distal part in early juvenile females (Fig. 5E, F–H, I). The heart remains close to the ciliary tract epithelium but becomes positioned at the trunk base as it elongates when the females grow (Fig. 5G).

### Gene expression in female stages with gut

In addition, we performed a differential gene expression analysis comparing female stages with a gut (4D, 5D larva and early female stage) against the same male stages (lacking a gut) and found 762 significantly upregulated genes in the females (Fig. S6). The gene-ontology (GO) enrichment analysis of these upregulated genes, including genes involved in angiogenesis (GO:0001525), revealed homeobox genes, such as *Homothorax* (*hth* or *Meis1* in vertebrate) and *hematopoietically expressed homeobox* (*hex*) (Figs. S6, S7). We also identified genes involved in tissue patterning like *Notch* (Fig. S7). Searching clustered genes (WGCNA), based on overexpressed gene in female stages with a gut, we found genes involved in sex differentiation such as *doublesex* *(Dmrt2)*(Fig. S7). Furthermore, we identified genes proposedly involved in excretion and filtration process, such as a urea transporter, (*DUR3*), Nephrin (*PGBM*), and an alkaline phosphatase (*PPBT*) (Fig. S7).

## Discussion

### Presence of a transient gut: a female character revealing genetically predetermined sex

We presented in this work the first description of a transient gut in the *Osedax* genus and linked that character to an early sexual dimorphism. The ciliated gut that appears in half of the 4D larvae is found to be a female character in *Osedax japonicus*. It persists during metamorphosis and develops concurrently with the elongation of the female body, until it disappears in the 2P juveniles around 11-day post-metamorphosis. Adult dwarf males exhibit a short ciliated spermioduct [23] but it only develops post-metamorphosis and has a different configuration than the female gut, hereby rejecting homology. Remarkably, this sexual dimorphism appears already in the larval stage of *Osedax*, further supported by our finding of sex biased gene expressions as early as in the 2D larvae. Such sexual transcriptomic signature preceding morphological dimorphism has recently been described for juvenile *P. dumerilii* [65], although not already in larval stages. The authors described sex-specific genes not necessarily related to reproduction [65]. Like our finding of a specific enzyme (*CA*) used in adult nutrition [17] and already found expressed in the larvae which indicates a pre-metamorphic onset of adult organogenesis. While gene expression preceding morphological divergence is anticipated, it had not been previously documented in *Osedax*.

**Fig. 6:**
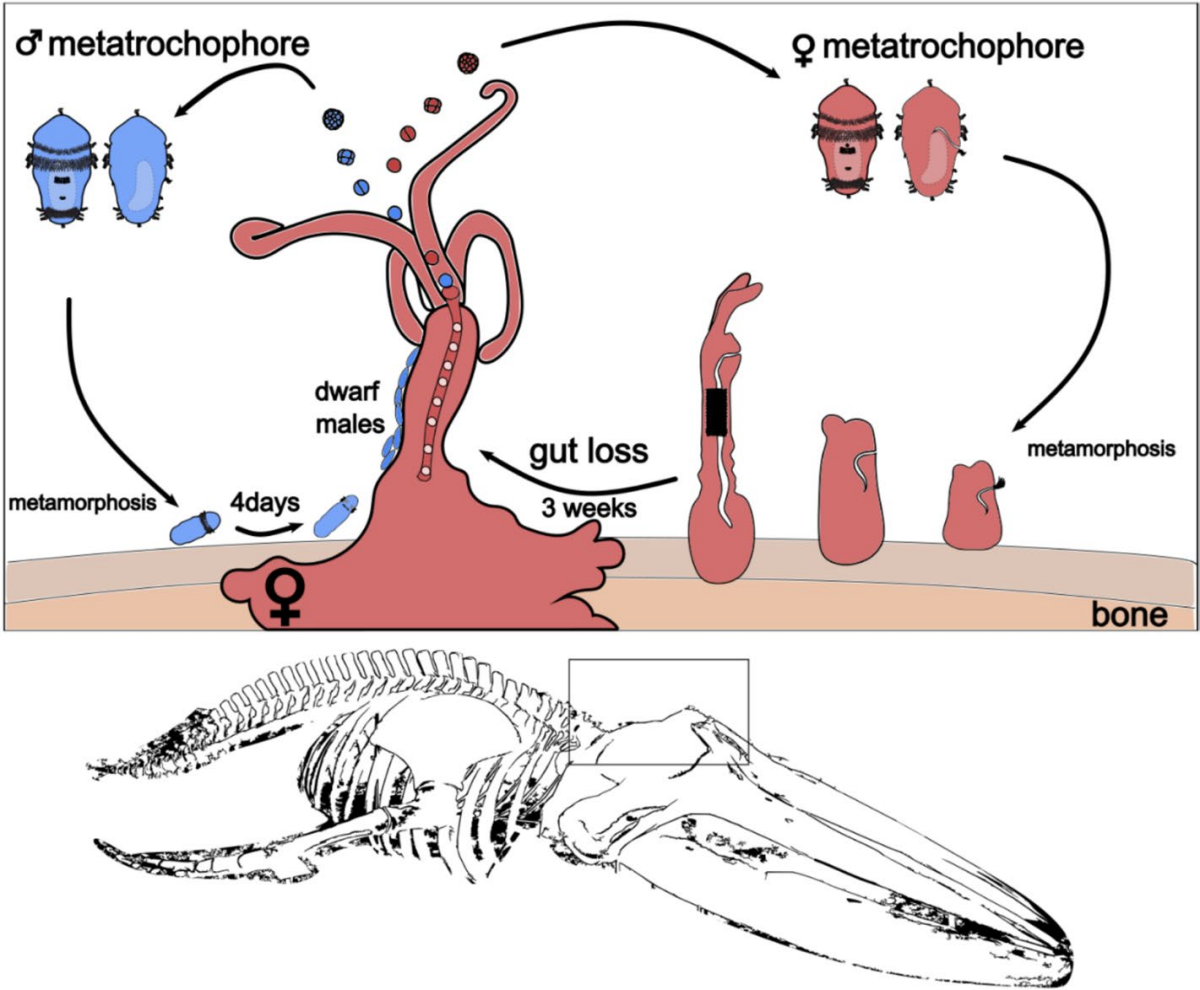
New life cycle hypothesis of the bone-eating worm *Osedax* having sexually predetermined larvae. Schematic drawing of *Osedax* development with sexually determined embryos developing into sexually determined larvae, female larvae having a gut, male larvae lacking one. Drawings are not scaled

The previously accepted hypothesis based on similar eggs size, seemingly similar morphology of the metatrochophore larvae, and successional male recruitment over time, suggested that larvae were sexually determined by environmental cues (bones for female, female presence for male) [2, 10, 27]. However, we, here, present evidence (presence of a gut, sex-specific gene expression in half of the competent larvae, 1:1 sex ratio) that the larvae are already sexually determined and that they differentiate pre-metamorphosis both at the molecular and the morphological level (Fig. 6).

### Is the transient gut in Osedax homologous with guts of other siboglinids?

The presence of a transient ciliated tract, with a ciliated mouth opening posterior to the prototroch, is already described in larvae of several other lineages of Siboglinidae (*Ridgeia sp.*, *Riftia pachyptila*, *Siboglinum poseidoni*, and *Oligobrachia mashikoi*) [7, 30, 33, 37, 38]. A through-gut with a ciliated anus is reported for the investigated vestimentiferans [31–33, 36, 42], while no anus develops in the frenulate *S. poseidoni* similar to our finding in female *O. japonicus* [30]. The position and ciliated structure seem similar, but contrary to *O. japonicus*, the gut in *S. poseidoni* larvae appears subsequent to (and not synchronous to) formation of chaetae, prototroch, and telotroch [22, 28, 30]. However, like in *O. japonicus*, the gut in vestimentiferans and frenulates persists until the juvenile stage. In *R. piscesae* a narrow ciliated through-gut is still present in the juvenile stage of ‘100-tentacles’ (trophosome, excretory organ) [33], and in *S. poseidoni* the gut is still present in juveniles with 4–6 opisthosome segments [30]. Comparing the exact staging across siboglinids is complex due to the divergent development and morphology of body regions. Nevertheless, we suggest homology of the transient gut in *O. japonicus* and other siboglinids based on structure, position, and development; all presenting a central ciliated tract appearing in the larval stage with a mouth opening posterior to the prototroch, and developing post-metamorphosis before disappearing in the late juvenile stages.

### Conserved but weak expression of gut markers

Previous reports of a ciliated gut in siboglinids were based on analyses of histology and TEM sections, but molecular studies were lacking. Investigating conserved gut and ventral markers we found that only *FoxA* and *GataB1* had a higher gene expression in females, yet only in few life stages. The pleiotropic functions of those genes (*FoxA*, *Gsc*, *GataB1*, and *WntA*) along with the transient nature of the gut may account for this observation. Notably, we report expression patterns in *O. japonicus* that demonstrate a relatively late onset, and more restricted territories of those markers compared to other annelids.

The expression of *FoxA*, classically found in cnidarian and bilaterian stomodeum and foregut (e.g., [50]), is conserved in *O. japonicus* female larvae, although with a late onset (3D stage) compared to *Owenia fusiformis* and *T. lageniformis* ([47, 52], Fig. S4). Consistent with *P. dumerilii* and *A. virens* [51], we observed *FoxA* signal in few ventro-lateral cells in the episphere of *Osedax japonicus*. Overall *FoxA* was expressed exclusively in female larvae, and a weak signal was found in the oral ectoderm and foregut, as well as in the juveniles’ midgut. *Gsc*, another highly conserved gut marker, was only transiently expressed in the foregut of *O. japonicus* 5D female larvae, compared to the longer and broader expression in the stomodeum and foregut of *P. dumerilii* and *C. teleta* [45, 48]. *WntA* expression is a reliable early ventral marker for the oral ectoderm region (4D stage) even if its territory extends towards the dorsal side, similar to previous observations in *P. dumerilii* [54]. In annelids, paralogs of *Gata4/5/6* are involved in the mesoderm or endoderm layer formation [46, 49]. We found similar lateral visceral band for *Ojap-GataB1* expression as of *GataB2* and *GataB3* in *C. teleta* [46]. However, we only observed a late midgut expression in *O. japonicus* juvenile females compared to a larval expression in *Owenia fusiformis*, *T. lageniformis* and *Chaetopterus sp.* [46, 47, 52]. Altogether, our results support that the transient ciliated tract in *O. japonicus* females is a rudimentary gut composed of a stomodeum, a foregut and a post-metamorphic midgut region.

### What are the possible roles of the transient gut?

Because no previous description of a transient gut has been made for *O. japonicus* larvae we questioned a potential role in feeding. *Osedax* larvae of *O. packardorum* (previously ‘orange collar’ sp.) and *O. rubiplumus* were characterized as lecithotrophic based on their resemblance to other vestimentiferan lecithotrophic larvae, their yolk content, and their absence of feeding apparatus or behavior [4, 21]. The rapid development of *O. japonicus*, with a 5-day period for a metatrochophore to become competent, the yolk content, the small size of the larvae, and the absence of feeding behavior [22] remain valid arguments for its lecithotrophic nature despite our discovery of a gut. In addition, the small size of the mouth opening and the very narrow gut lumen, as well as the absence of labelled bacteria suggest the transient gut is unlikely to perform any feeding function.

The symbiont horizontal transfer in Vestimentifera and Frenulata was initially hypothesized to occur through the larval transient gut; however, studies have evidenced an epidermal acquisition [30–33, 42]. In *Osedax*, epidermal acquisition was likewise previously assumed, notably since no transient gut was known [15, 17, 20, 43]. The discovery of a female transient gut made us question its role in symbiont acquisition. However, we did not detect bacterial 16S signal in the gut dermis; instead, we observed signal in the root of the 2P females, further supporting epidermal acquisition of symbionts as the common mechanism of Siboglinidae.

Given that the gut in vestimentiferans, frenulates, and *Osedax* does not support feeding function in the lecithotrophic larva, nor symbiont acquisition, and is lost in adults, it is reasonable to hypothesize that it represents a vestigial morphological trait that has not yet been lost. However, unlike other siboglinids, *Osedax* species (except *O. Priapus*, [64]) exhibit strong sexual dimorphism, and paedomorphic dwarf males. In studies of vestimentiferan and frenulate gut systems, there was no report of specimens lacking a gut [7, 30–33, 36–42], suggesting that the gut is not sex-specific in those species. The persistence of this seemingly “non-functional” organ in females compared to its loss in males (larvae and adults) supports ontogenetic constraints, at minimum, or potentially a specific function, related to the macroscopic size and biology of female *O. japonicus* and other siboglinids.

The blood vascular system and the ‘heart’, the most muscularized and major pulsatile part of the dorsal blood vessel, are among the ‘adult’ organs lost in the paedomorphic male *Osedax*’s [13, 14, 22, 64]. The gut could play a role in the heart fate patterning and/or as an exchange surface, as suggested both by its persistence until the juvenile 2P stage and by the proximity of the gut to the ‘heart body’, proximity also previously reported in *Ridgeia sp.* [31] and *S. poseidoni* [30]. Among the potential juxtacrine signaling system that could be involved in that patterning, the *Notch* pathway was the most complete pathway (including *Notch*, *Delta*, and *Jagged*) found to be overexpressed in females. It would be valuable to investigate the implication of the *Notch* pathway in *O. japonicus* vascular system development, notably because its components exhibit localization in *P. dumerilli*’s stomodeum [66] and in *C. teleta* larva’s foregut [67].

An alternative hypothesis for the gut function is as an additional ‘exchange surface’ and potentially an excretory organ. The gut remains present until the development of the second pair of palps and the vascularized palp pinnules, that are the major exchange surface, allowing O_2_ uptake and sulfide release or detoxification [13]. The gut could also function as an additional excretory organ similar to enteronephridia found in some small-sized annelids (e.g., [68]); indeed, protonephridia have been described in the 5D larva of *O. japonicus*, but no nephridia have yet been identified in the adult female [22]. Katz and Rouse [14] discussed the potential presence of nephridia in the posterior part of the adult female, finding a closed coelomic pouch. Still, they also highlight the absence of ciliation and the unconventional position of that potential nephridia, compared to the anterior trunk part position in other siboglinids. To date, no further evidence of nephridia in *Osedax* females has been reported. Among the genes found overexpressed in females compared to males, we found an alkaline phosphatase (*PPBT*) used as a marker for secretory epithelia such as nephridial structure [69]. Elucidating its localization could provide insight into a putative involvement in a resorptive function of the gut and of the excretory mechanism in *O. japonicus* adult. Nevertheless, not all *Osedax* species possess elaborated palps or palps bearing pinnules [16]. Investigating the presence of a gut both in larvae and early females of *Osedax* with diverse appendages (nude palps, pinnulates palps, no palps) [11] would help resolve a potential function in exchange mechanism.

## Conclusion

Altogether, our findings demonstrate the presence of a gut in half of the larvae of an *Osedax* species, making it the first sexual dimorphism character ever described at the larval stage for the genus. We showed both morphologically and at the gene expression level that larvae are already sexually differentiated, refuting previous hypothesis about *Osedax* sex determination (Fig. 6). In line with most recent work done on other siboglinids, we showed that the function of the gut was unlikely to be the uptake of the symbionts. Although a transient gut had already been described in other representatives of Siboglinidae, only the strong sexual dimorphism between adults of *O. japonicus* allowed us to draw a functional hypothesis. We propose that the transient gut is involved in patterning the formation of the female vascular system and more specifically the ‘heart’ and/or can act as an exchange surface prior to the development of the pinnules and the ventral palps.

## Supplementary Information

Supplementary Material 1

Supplementary Material 2

Supplementary Material 3

Supplementary Material 4

Supplementary Material 5

## Data Availability

Sequencing data supporting the conclusions of this article are available on BioProject repository (PRJNA1088276, PRJNA1217611) and in the additional files. Scripts are available on a GitHub “Ojap_Gut” repository. HCR scans used in figures are available on the public ERDA archive (10.17894/ucph.6dcc2b2f-4e34-49ff-845a-723543d095ea) and remaining IF full scans are available upon request.

## Abbreviations

5D: 5 Days
2P: 2-Palps
CA: Carbonic anhydrase
CLSM: Confocal laser scanning microscopy
dpi: Days post-induction
Dmrt2: Doublesex
ER: Oestrogen receptor
FoxA: Forkhead A
Glb3: Extracellular globin 3
GO: Gene ontology
Gsc: Goosecoid
HCR: Hybridization chain reaction
hex: Hematopoietically expressed homeobox
hpc: Hours post-cleavage
hpi: Hours post-induction
hth: Homothorax
IF: Immunofluorescence
WMISH: Whole mount in situ hybridization
WGCNA: Weighted gene co-expression network analysis
Wnt: Wingless
Zan: Zonadhesin
