# Supplementary material for: Predetermined sex revealed by a female transient gut in non-feeding larvae of Osedax (Siboglinidae, Annelida)
Source: EvoDevo. 2025 Aug 26;16:15. doi: 10.1186/s13227-025-00251-9 (PMC12382133; doi:10.1186/s13227-025-00251-9)
Supplement: Supplementary file 1 — Supplementary Material 1 [file 13227_2025_251_MOESM1_ESM.docx]

**Predetermined sex revealed by a female transient gut in non‑feeding larvae of *Osedax* (Siboglinidae, Annelida)**

Alice Rouan,^1^* Norio Miyamoto,^2^ Katrine Worsaae^1^*

^1^Marine Biological Section, Department of Biology, University of Copenhagen, Universitetsparken 4, 2100 Copenhagen Ø, Denmark.

^2^X-STAR, Japan Agency for Marine-Earth Science and Technology, Yokosuka, Japan.

https://doi.org/10.1186/s13227-025-00251-9

**Supplementary material**


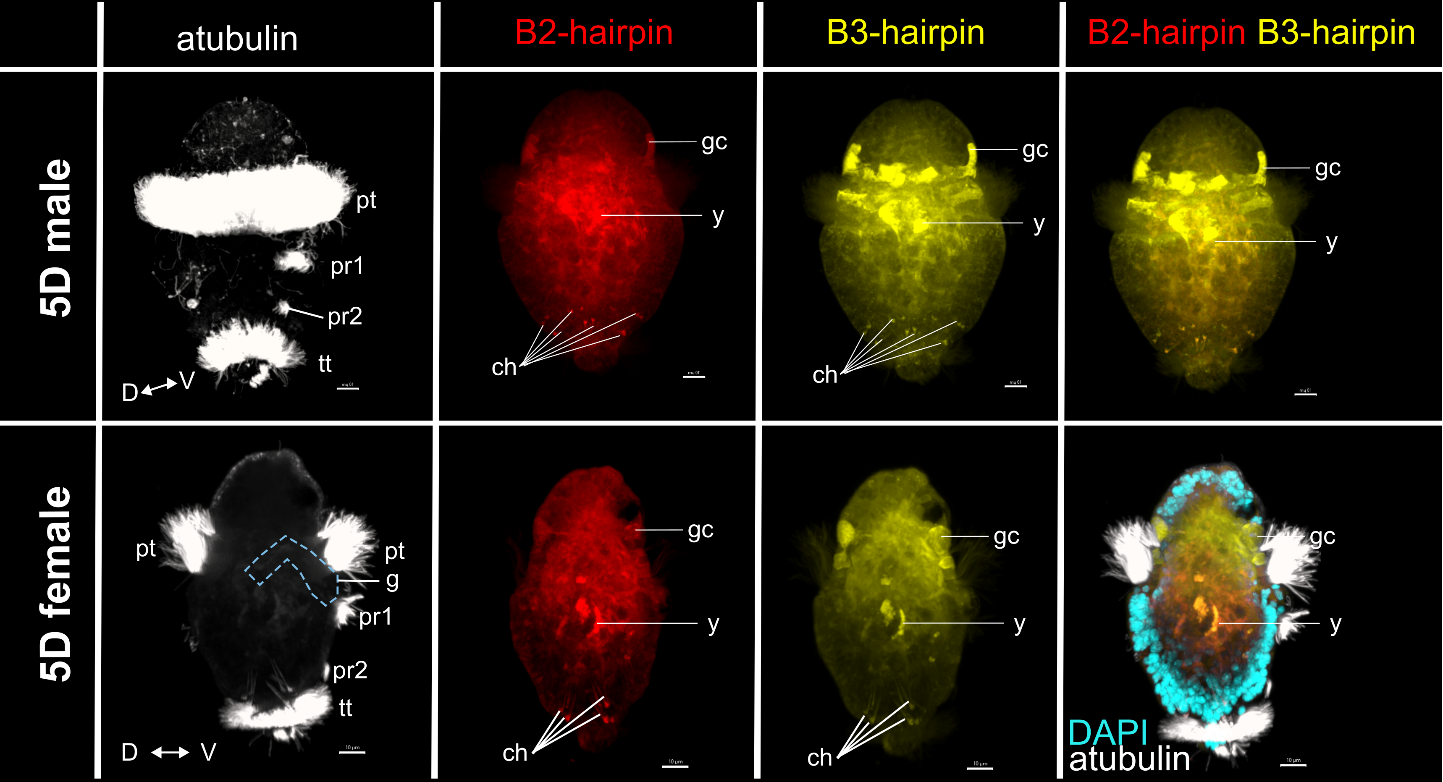

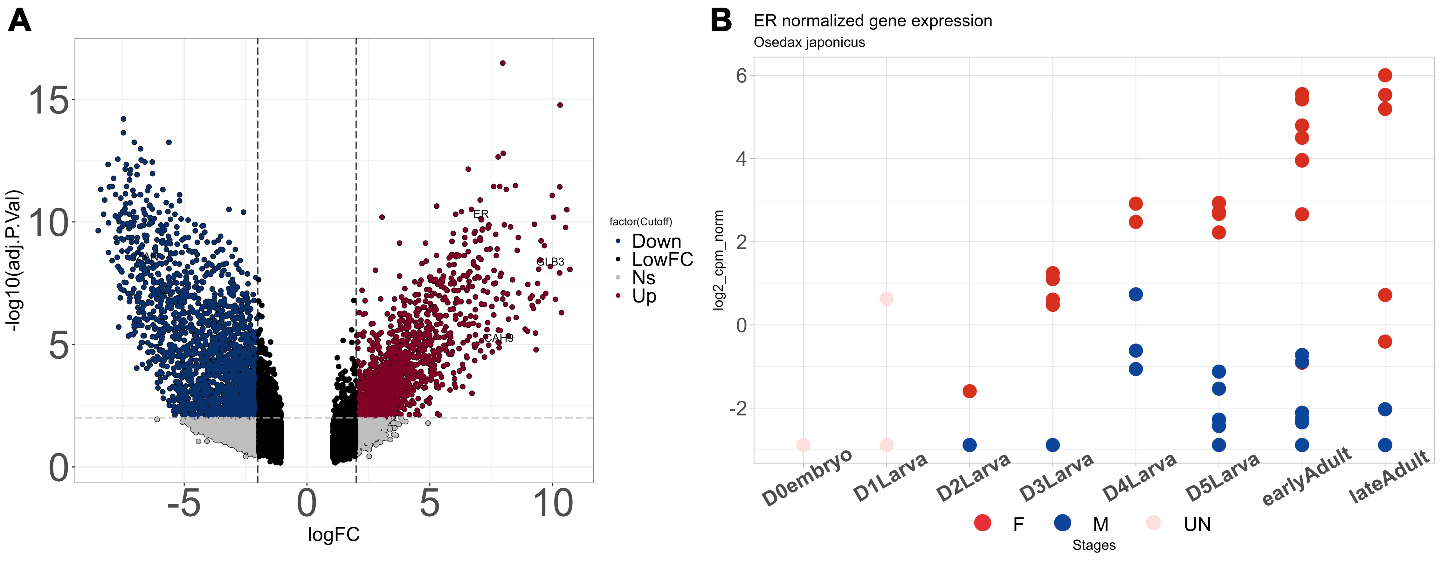
**Fig. S1**: **A** Volcano plot of differential gene expression (logFC=|2|, adj.p.value<0.01) between female (juvenile and adult) and male (juvenile and adult) stages**. B** Individual normalized cpm expression of oestrogen receptor (ER) displaying sex with colors, female (*red*), male (*blue*) and undetermined (*pink*).

**Fig. S2:** Negative control of whole mount in situ-HCR using B2-hairpins (red) and B3-hairpins (yellow) alone combined with anti-acetylated α-tubulin immunostaining (*white*) and nuclei stain (DAPI, *cyan*) in 5-day old male and female larvae. Stain shows the background of pigmented, potentially glandular, cells located beneath the prototroch, and of the yolk in the centre of the animal as well as the chaetal autofluorescence signal.


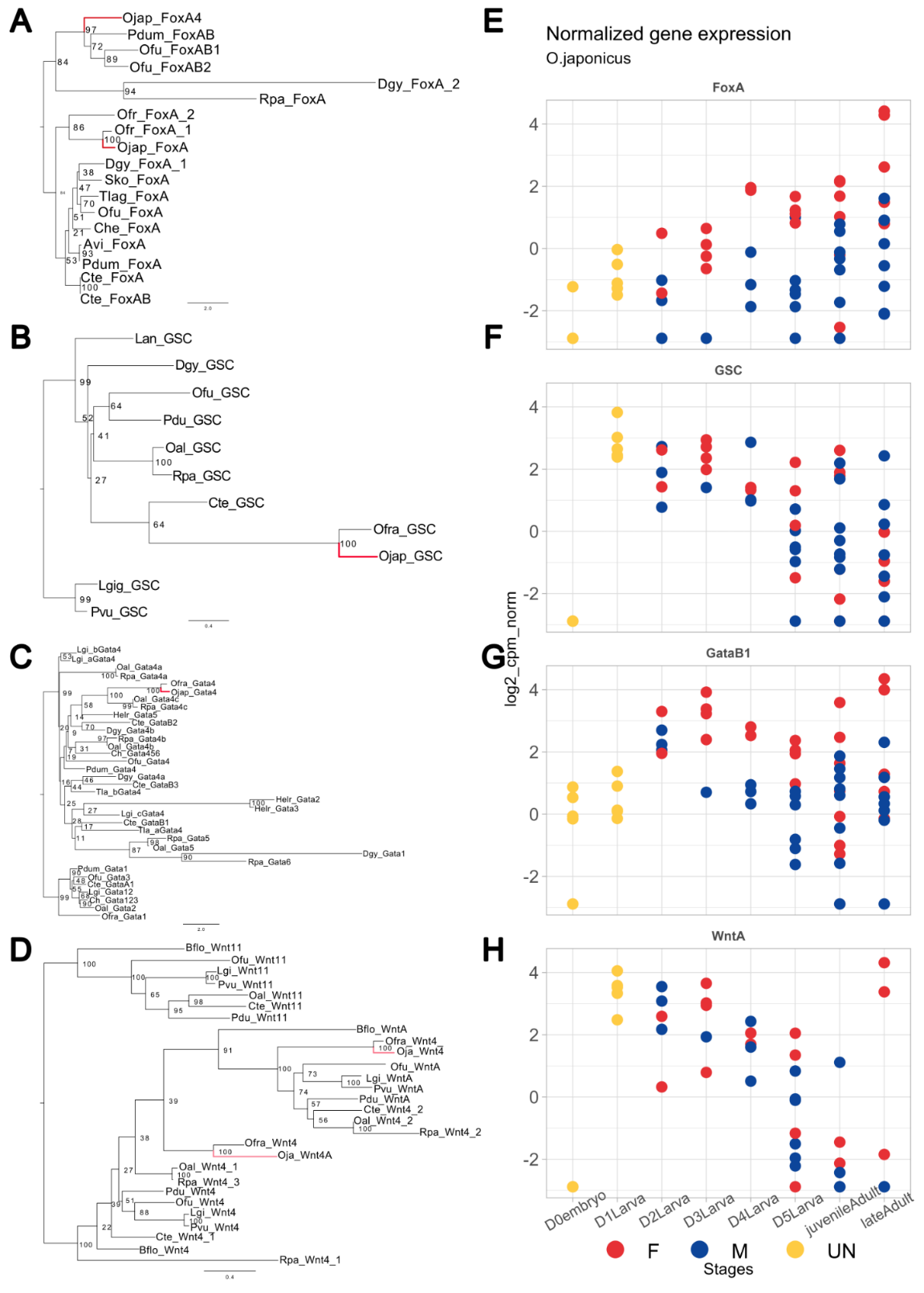


**Fig. S3:** Gut marker orthology analyses and expression levels. **A-D** phylogenetic trees of *FoxA*, *Gsc*, *Gata* and *Wnt* (*WntA*, *Wnt1* and *Wnt4*) inferred from maximum likelihood analysis, *O. japonicus* branches are labelled in red, data are available in Table S3. **E-H** individual gene normalized cpm expression of temporal stages (*D0embryo* early embryo, *D1-D5Larva* 1 day old to 5-day old larva, *juvenile* and *adult*) colored by sex with female (*red*), male (*blue*) and undetermined (*yellow*) specimens.


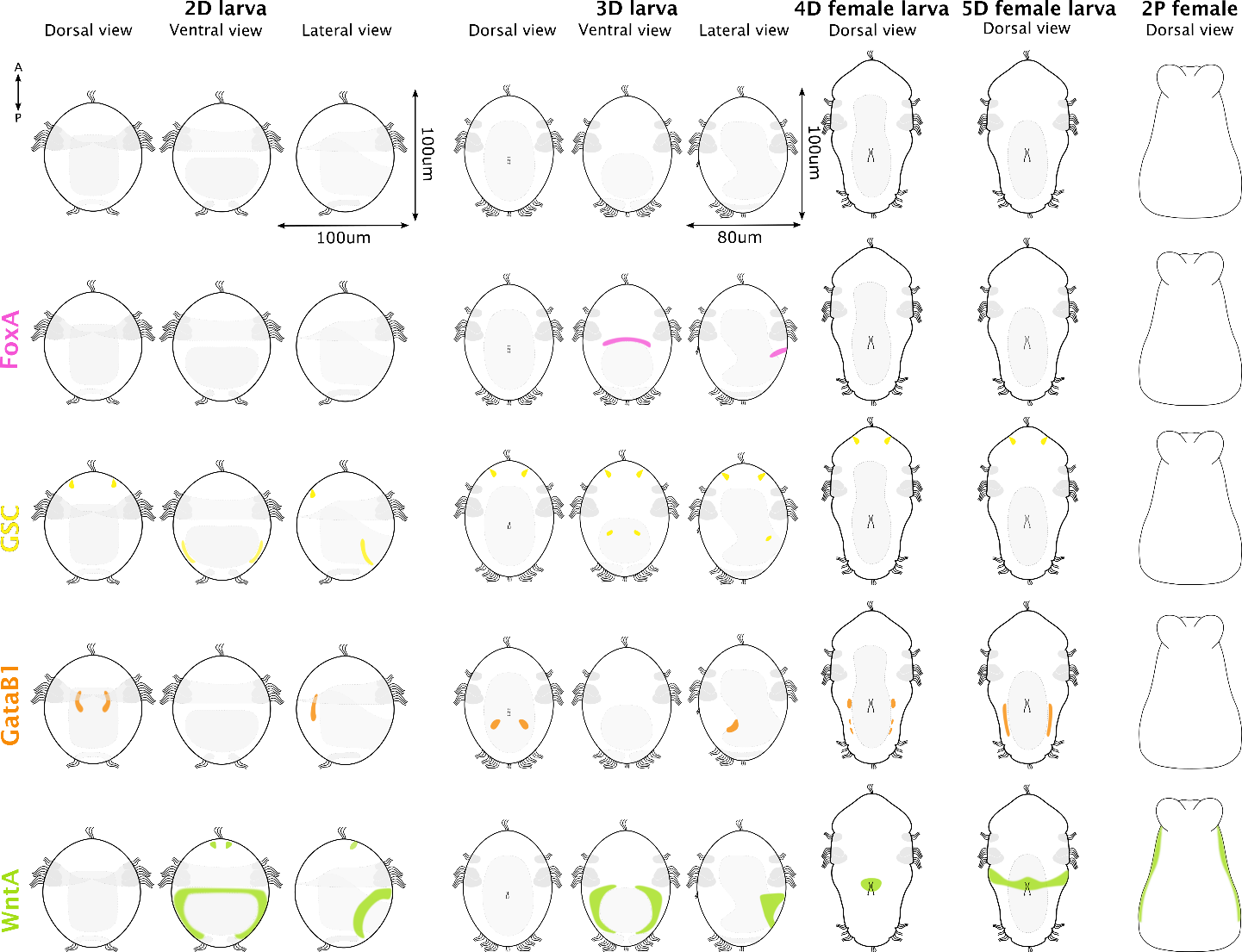


**Fig. S4:** Drawing of 2D-3D larvae and dorsal view of 4D-5D larvae and 2P females showing *FoxA* (*pink*), *Gsc* (*yellow*), *GataB1* (*orange*) and *WntA* (*green*) expression pattern, the sex could not be assessed for the 2D and 3D stages due to the lack of distinct morphological sexual dimorphism.


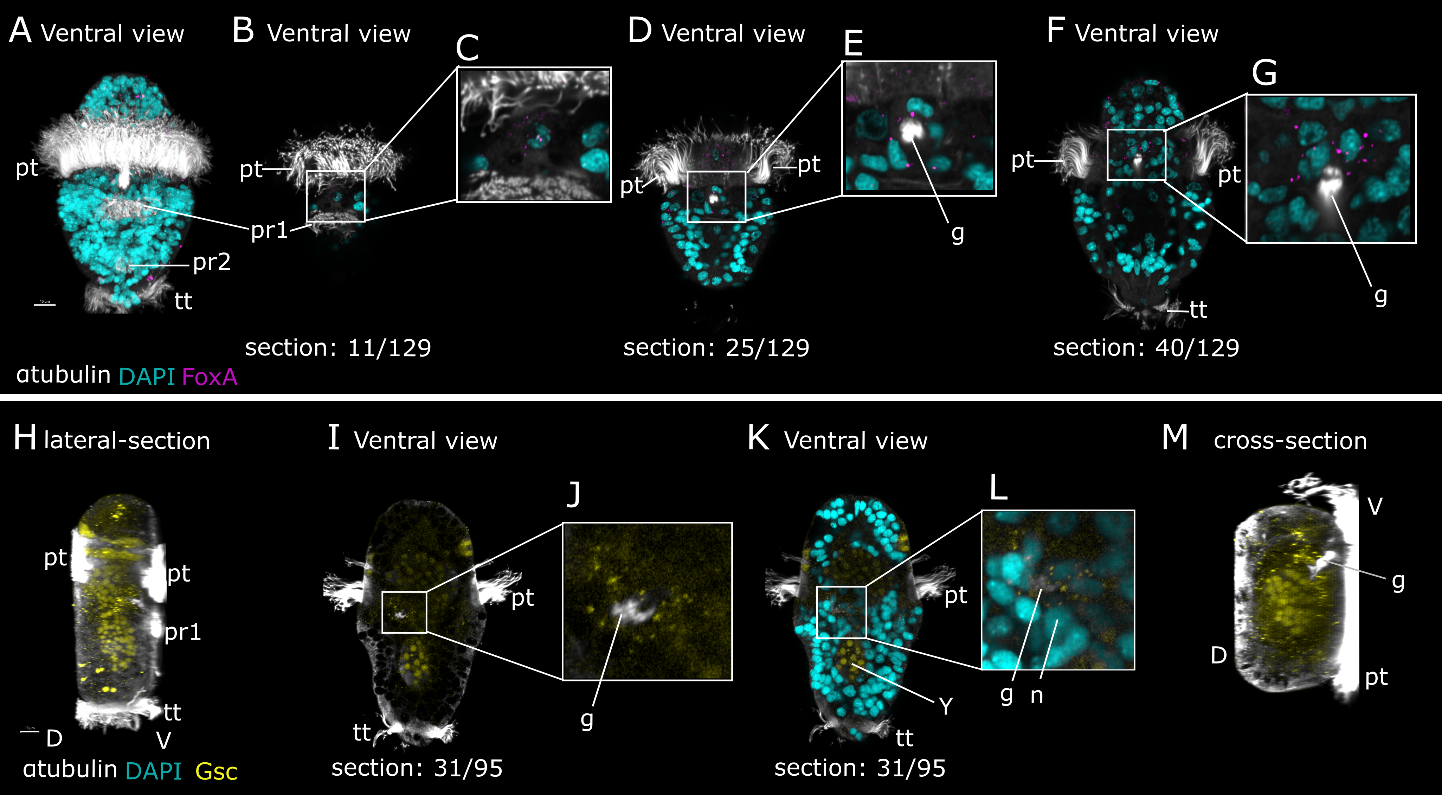


**Fig. S5:** Whole mount in situ-HCR of *Foxa* (pink) and *Gsc* (yellow) coupled with acetylated α-tubulin immunostaining (*white*) and nuclei stain (DAPI, *cyan*) in 5-day old female larvae. **A** maximum projection of ventral part of 5D larva, ventral view. **B** single sub-ventral section (no. 11/129) and **C** close-up of gut region, showing *FoxA* signal (pink) in a gut lining cell (nuclei, cyan). **D** single sub-ventral section (no. 25/129) and **E** close-up, showing signal in cells lining the gut. **F** single sub-ventral section (no**.** 40/129) and **G** close up, showing the curvature of the gut and *FoxA* signal in surrounding cells. **H** maximum projection of median part of 5D larva, lateral view (dorsal to the left, ventral to the right), showing *Gsc* (*yellow*) signal. **I-L** single sub-ventral section (no. 31/95) and close up showing *Gsc* expression inside the ciliary gut cells. **M** cross section of the prototroch region showing gut (dorsal to the left, ventral to the right). *D* dorsal, *g* gut, *pt* prototroch, *pr1/2* paratroch 1/2, *tt* telotroch, *V* ventral, *y* yolk.

**
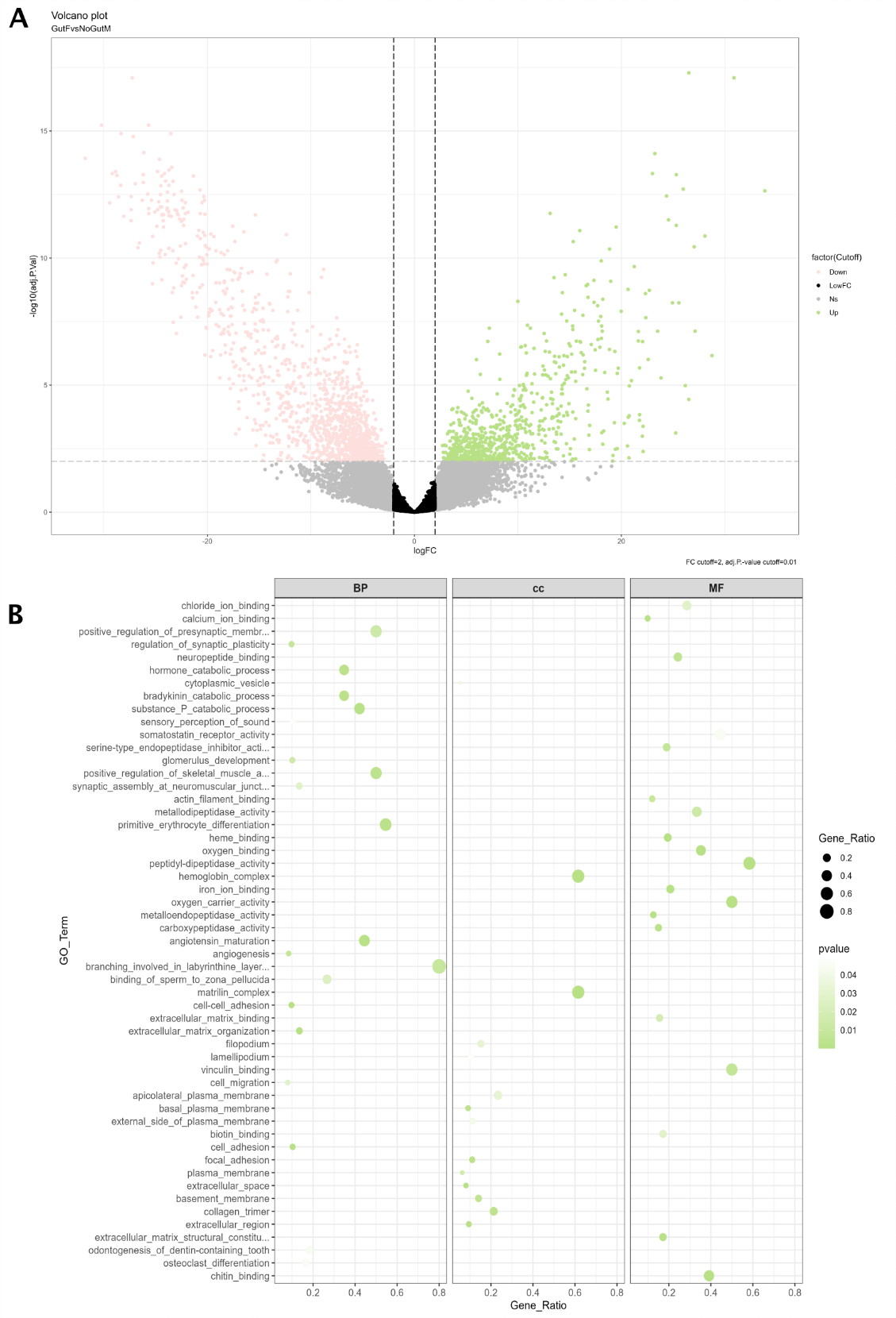
Fig. S6:** Differential gene expression in female stages with gut. **A** Volcano plot of differentially expressed genes between (4D-5D larvae, juvenile and adult) females and identical male stages (logFC=|2| and adj. pvalue<0.01). **B** Go-Term enrichment plot of Biological Process (BP), Cellular Component (CC) and Molecular function (MF) of the 762 upregulated genes in females (4D-5D, juvenile) compared to male.

**
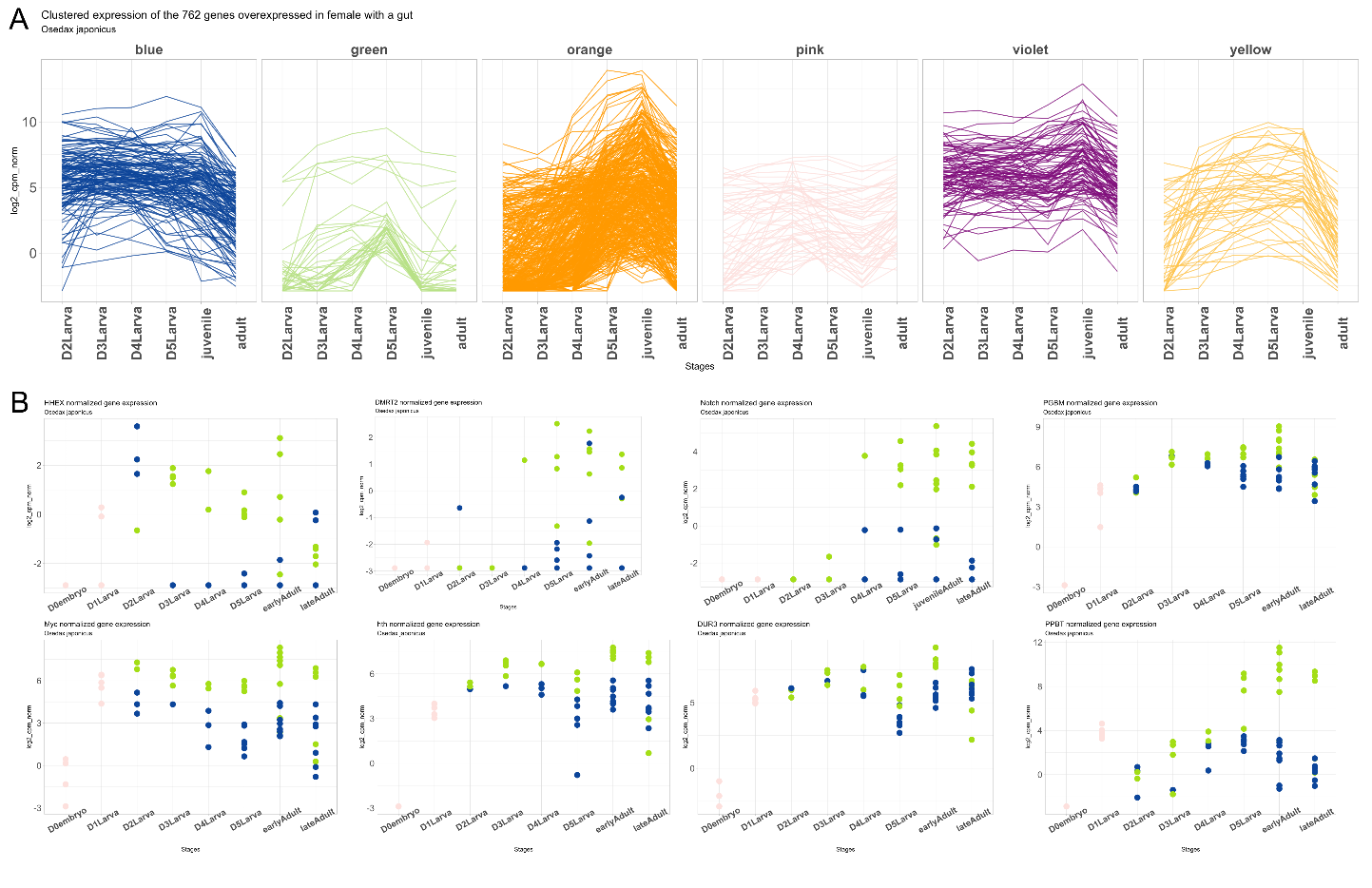
Fig. S7:** Clustering of overexpressed genes in females with a gut. **A** WGCNA of the 762 upregulated genes in the identified female stages with a gut (2D-5D, juvenile) displayed with a log2 scale of normalized cpm read counts. **B** Individual gene normalized cpm expression of *Hhex* (Ojap_80468_c0_g1), *Dmrt2* (Ojap_1030_c1_g1), *Notch* (Ojap_16025_c0_g1), *PGBM* (Ojap_1688_c0_g1), *Myc* (Ojap_13454_c0_g1), *hth* (Ojap_103_c0_g2_i2), *DUR3* (Ojap_1795_c0_g1) and *PPBT* (Ojap_14461_c3_g2) of temporal stages (D0embryo, 1D-5D larva, juvenile and adult) colored by sex female (*green*), male (*blue*) and undetermined (*pink*). Juveniles are also referred to as early adults.
